# Complex artificial intelligence models for energy sustainability in educational buildings

**DOI:** 10.1038/s41598-024-65727-5

**Published:** 2024-07-01

**Authors:** Rasikh Tariq, Awsan Mohammed, Adel Alshibani, Maria Soledad Ramírez-Montoya

**Affiliations:** 1 Institute for the Future of Education, Tecnologico de Monterrey, Ave. Eugenio Garza Sada 2501, 64849 Monterrey, NL Mexico; 2 Architectural Engineering and Construction Management Department, King Fahd University of Petroleum and Minerals, 31261 Dhahran, Saudi Arabia; 3 Interdisciplinary Research Center for Smart Mobility and Logistics, King Fahd University of Petroleum and Minerals, 31261 Dhahran, Saudi Arabia; 4 Interdisciplinary Research Center of Construction and Building Materials, King Fahd University of Petroleum and Minerals, 34463 Dhahran, Saudi Arabia; 5 EGADE Business School, Tecnologico de Monterrey, 64849 Monterrey, NL Mexico

**Keywords:** Higher education, Educational innovation, Educational buildings, Energy consumption, Machine learning, Energy science and technology, Mathematics and computing

## Abstract

Energy consumption of constructed educational facilities significantly impacts economic, social and environment sustainable development. It contributes to approximately 37% of the carbon dioxide emissions associated with energy use and procedures. This paper aims to introduce a study that investigates several artificial intelligence-based models to predict the energy consumption of the most important educational buildings; schools. These models include decision trees, K-nearest neighbors, gradient boosting, and long-term memory networks. The research also investigates the relationship between the input parameters and the yearly energy usage of educational buildings. It has been discovered that the school sizes and AC capacities are the most impact variable associated with higher energy consumption. While 'Type of School' is less direct or weaker correlation with 'Annual Consumption'. The four developed models were evaluated and compared in training and testing stages. The Decision Tree model demonstrates strong performance on the training data with an average prediction error of about 3.58%. The K-Nearest Neighbors model has significantly higher errors, with RMSE on training data as high as 38,429.4, which may be indicative of overfitting. In contrast, Gradient Boosting can almost perfectly predict the variations within the training dataset. The performance metrics suggest that some models manage this variability better than others, with Gradient Boosting and LSTM standing out in terms of their ability to handle diverse data ranges, from the minimum consumption of approximately 99,274.95 to the maximum of 683,191.8. This research underscores the importance of sustainable educational buildings not only as physical learning spaces but also as dynamic environments that contribute to informal educational processes. Sustainable buildings serve as real-world examples of environmental stewardship, teaching students about energy efficiency and sustainability through their design and operation. By incorporating advanced AI-driven tools to optimize energy consumption, educational facilities can become interactive learning hubs that encourage students to engage with concepts of sustainability in their everyday surroundings.

## Introduction

Energy consumption of constructed facility has a significant impact on the environment. The world is currently experiencing significant fluctuations in the energy landscape, which has far-reaching implications for many aspects of life on a daily basis. Furthermore, global energy demand has risen by 0.9% to 120 millions tons of oil equivalent (Mtoe)^1^. The International Energy Agency anticipates that by 2021, the buildings and construction industry will account for more than one-third of global energy demand. Furthermore, this sector is projected to contribute to approximately 37% of the carbon dioxide emissions associated with energy use and procedures^2^. The impact of this dioxide emissions on the environments, social, and have recently increased, emphasizing the growing importance of sustainable development. Meanwhile, there is a growing demand for building energy services. Therefore, there is a critical need to develop effective strategies for energy planning and prediction. This not only ensures the future progress of the built environment, but also enhances energy efficiency, conserves resources, and promotes sustainable development^3,4^. Efficient strategies involve accurately forecasting building energy usage to effectively oversee and conserve energy within buildings.

Educational institutions, including schools, represent a major part of built environment facility that play a crucial role in shaping the future of society through knowledge dissemination and fostering intellectual growth. However, schools represent significant energy consumers, with diverse facilities requiring heating, cooling, lighting, and other services. The efficient management of energy resources in educational buildings is not only essential for cost savings but also aligns with broader sustainability goals aimed at reducing carbon emissions and mitigating climate change impacts^5^. The energy usage in school buildings is influenced by various factors, including their location, size, number of occupants, age, and extent of air conditioning. Accurately predicting energy usage is essential for the effective functioning of contemporary electrical grids^6,7^. Numerous research studies have emphasized the importance of precise electricity forecasting. Accurate and reliable estimates of electricity consumption are critical for planning future electricity generation systems to meet the growing demand for electrical energy^8,9^. Understanding the variations in building energy usage allows to development of focused and efficient energy conservation strategies^10^. Comprehensive knowledge of building energy consumption prediction is essential for developing innovative approaches such as demand-side management plans^11^, intelligent control systems^12^, and fault detection and diagnosis methods^13^. These methods use predictive analysis to improve energy efficiency, reduce waste, and ensure that building systems run smoothly. These systems encourage both energy conservation and building infrastructure efficiency by determining potential energy-saving possibilities and resolving inefficiencies in operation^14^. Studies have shown that even slight improvements in estimating building energy usage can result in significant energy savings^15^. Managers of buildings and users may adopt better decisions and take proactive steps to increase energy efficiency by precisely predicting energy use patterns. This could involve changing HVAC configurations, improving the schedule of lighting, installing energy-efficient devices, and applying behavioral adjustments to help achieve energy-saving targets^16^.

However, accurate prediction of energy consumption is difficult due to a variety of unpredictable situations or noisy data disorders, and the methods used frequently produce inaccurate projections. Thus, this field requires additional work and attention, particularly from governmental agencies. Consequently, this paper investigates number of artificial intelligence-based models to estimate the energy consumption of school buildings. These models include decision trees, K-nearest neighbors, gradient boosting, and long-term memory networks. The investigated algorithms are developed and validated using real data collected for educational buildings; school. In addition, different statistical tests are conducted to ensure the quality of data. The models are expected to contribute to effective resource management, environmental sustainability, educational opportunities, operational efficiency, and regulatory compliance. Educational institutions can create greener schools while saving money by accurately estimating energy needs and implementing consumption-reduction strategies.

## Literature review

### Sustainable school buildings contributing towards educational innovation

In the context of complexity, it is important to look for innovative options that support institutions to become more sustainable in terms of energy. Purnell et al.^17^, implemented plans involving the academic and social community, where they learned about innovation, leadership, coal mining, greenhouse problems, energy audits, alternative energy and promotion of energy efficiency practices in the school and community. Designing the construction or restructuring of school buildings is vital to improve energy consumption^18^. New solutions are needed, hence Zeiler and De Waard^19^ presented alternatives for sustainable schools through ‘Plus Energy Schools’, the authors based on a study comparing passive house schools, near-zero energy schools and additional energy schools, where they evaluated the indoor air quality and comfort of some of the schools measured, with a view to contribute to the challenging design towards more sustainable schools. Improving school infrastructure requires attention to the principles of sustainable development.

The designs of school institutions require new ecological, sustainable and healthy practices that accompany educational innovation practices. Educational innovation integrates inputs of processes, products, services or knowledge with a view to improving complex environments^20^ and in institutions, the use of new technologies, evolving sustainable design practices and innovative ‘green’ building materials should be promoted for each new project to be reflected in constantly improving energy efficiency results^21^. In parallel to sustainable care, it is relevant that institutions are energy neutral, energy positive and provide comfort to stakeholders (students, teachers, managers, parents) in learning environments^22^. Zhang et al.^23^ provided an energy prototype with sustainable strategies and energy-saving technologies, which can reduce energy consumption in school facilities as well as improve the indoor environment. Sustainable design and construction strategies that combine high levels of energy efficiency, performance standards and indoor environmental quality should integrate experimentation and contribute to formulating innovative sustainable building strategies^24^. Innovation and sustainability are substantial elements for educational institutions in the context of constant change.

### Complex artificial intelligence modelling through machine learning methods in educational buildings

In Artificial intelligence has been of value in modelling solutions in various sectors. For example, in river studies, Msaddek et al.^25^ employed artificial intelligence to reduce potential uncertainties using the Unsupervised Multi-Frameworks Technique and Fuzzy Membership Framework and supervised learning based on the Multi-Model Approach and Gene Expression Programming. Similarly, Tao et al.^26^ applied artificial intelligence models for suspended river sediment prediction to provide an updated description of the most recent and relevant AI-based applications for modelling sediment transport in watershed systems. A combination of water management combined with socio-technical systems is presented by Baki et al.^27^, assessing the dynamic nature of socio-economic variables in order to test the effectiveness of different policies, such as awareness campaigns, and dynamically simulate the subsequent response of the urban water system over time. In the financial sector, Méndez-Suárez et al.^28^ integrated artificial intelligence for automated financial advice in the copper market with promising results, both in terms of statistics and trading metrics. Modelling through machine learning provides opportunities for various sectors, including the education sector.

Educational buildings also benefit from complex artificial intelligence modelling. Reddy et al.^29^ conducted a plug load study in educational buildings using machine learning algorithms to monitor their energy consumption where they applied machine learning techniques. López-Pérez & Flores-Prieto^30^ studied energy savings in an air-conditioned educational building in the tropical climate of Aw in relation to annual cooling load and degree-days, with adequate comfort levels following the adaptive thermal comfort approach. Comfort temperature modelling was performed using fuzzy logic, artificial neural networks, adaptive neuro-fuzzy inference system and a local linear model. Hosseini et al.^31^ modelled, analysed and optimised an integrated energy system for the provision of triple loads of an educational building using artificial intelligence, showing that the dynamic load pattern provides the highest energy efficiency and the lowest total cost rate. Lee & Zhang^32^ provide a new artificial intelligence of things (AIoT)-based framework for predicting multidimensional indoor environment quality (IEQ) conditions that provides information on IEQ conditions and their potential impacts on student well-being, thus facilitating the future development of climate-adaptive, data-driven, human-centric educational facilities. Artificial intelligence modelling through machine learning methods in educational buildings has a high potential to improve educational environments, their sustainability and energy efficiency.

### Summary of literature review and research contribution

Since 2002, government agencies, including the European Union, have assessed building energy efficiency to forecast energy consumption^33^. The fundamental purpose of energy prediction is identifying and forecasting potential enhancements to optimize energy consumption within a building. There are several energy prediction methodologies available, each with its own set of benefits and drawbacks^34^. Previous studies provided a review of energy prediction methodologies used to forecast building energy performance^35–37^. Chae et al.^38^ used an Artificial Neural Network (ANN) model with a Bayesian regularization technique to predict the short term energy usage of building. The authors analyzed several design criteria to determine the most effective structure for the prediction model. Biswas et al.^39^ built and verified an ANN model to meet the non linear difficulty of acquiring data on energy use while providing reliable computation of large dynamical datasets. Deb et al.^40^ also introduced an ANN for estimating the consumption of energy from both night and day cooling demands in educational facilities. The authors separated the energy usage data into various kinds of inputs.

Yan et al.^41^ proposed a hybrid model based on a neural network to forecaste energy consumption in individual households. This model was created primarily to address issues related to irregular human behavior and univariate datasets found in single household energy consumption prediction. Zhong et al.^42^ developed a support vector machine to address issues with the accuracy of forecasting related to the significant non-linearity between both outcomes and inputs in energy usage prediction models. Wang^43^ and Tabrizchi^44^ also used a self-adaptive multiverse optimization to enhance the accuracy of prediction and tune the features of the support vector machine. Meanwhile, Iwafune et al.^45^ used regression models to forecast the electricity usage in 50 households. Albuquerque et al.^46^ employed standardized machine-learning algorithms to forecast Brazil's consumption of electricity. The findings showed that random forests provided forecasts. In addition, Dong et al.^47^ introduced a technique for forecasting energy consumption specific to building operations across multiple timeframes, employing pattern classification and integrated learning. They utilized random forests to assess the significance of input features in the model and conducted correlation analysis to explore the connection between the input parameters and output. The results showed that the integrated consumption of energy forecasting approach with pattern categorization outperformed the unclassified integrated energy consumption prediction model.

Cao et al.^48^ presented an integrated model for predicting energy consumption that uses spatial features taken from time-series information to estimate short-term energy usage in higher education organizations. They used the collaborative game theory SHAP approach to evaluate the influence of features on model performance, using ablation analysis to identify the optimum amount of features needed. Faiq et al.^49^ utilized LSTM to estimate energy usage in institutional buildings. The authors evaluated the model's performance against SVM and Gaussian process models. Álvarez et al.^50^ studied 453 homes in Spain to estimate their U-opaque rating. Several ANN structures were developed and evaluated on actual recorded data, with a Pearson correlation factor of 0.967. Beccali et al.^51^ proposed using artificial neural networks to anticipate building energy efficiency in buildings in Italy. Ahmad et al.^52^ investigated the performance of random forest and ANN models for predicting HVAC power usage in hostels. The findings indicated that ANN outperformed random forest slightly. Martellotta et al.^53^ employed to estimate heat energy usage, training the network on EnergyPlus-generated simulated data. Williams and Gomez^54^ studied 426,305 single-family dwellings to anticipate monthly energy usage using building features and monthly weather data. Sun and Han^55^ provided an ANN model that took into account a variety of input factors such as orientation, window size, windows per bay, number of floors, and floor height. Similarly, Wong et al.^56^ utilized ANN to forecast the overall energy usage, taking into account nine weather-related input factors, four-building envelope variables, and one-day category.

Moreover, Catalina et al.^57^ constructed regression models to forecast the monthly heat demand of homes in France. The study incorporated factors such as the building's structural U-value, window-to-wall ratios, and the construction shape factor. Al-Rashed and Asif^58^ investigated a variety of variables that impact the use of energy in residential buildings, including building envelopes, weather patterns, cooking appliances, dwelling types, and air conditioning systems. the findings indicated the use of double-glazing installations and air conditioning with mini-split systems. However, the authors point out a limitation: the inability to evaluate architectural design in order to reduce energy costs during the early design phase. Abdel-Aal et al.^59^ developed a model to predict electric power energy consumption in Saudi Arabia's Eastern Province that takes into account conditions as well as economic and population aspects. Nasr et al.^60^ proposed an ANN forecasting approach for estimating electric energy consumption in Lebanon using weather variables and time series data. Meng et al.^61^ used a hybrid approach to forecast the increasing trend for electrical consumption of energy. However, one disadvantage of this methodology is that it fails to include architectural aspects and design integration when estimating monthly electric energy demand. Karatasou et al.^62^ used statistical analysis tools to investigate potential improvements in ANNs accuracy for forecasting the consumption of building energy. Mena-Yedra et al.^63^ developed a short term ANN approach for estimating energy load at an industrial buildings, resulting in timely solutions.

In addition to the studies mentioned above, Somu et al.^64^ presented an integrated approach for forecasting residential energy consumption that combines long short-term memory and neural network. The findings demonstrated promising results in predicting previously difficult energy consumption patterns. Fath U Min Ullah et al.^65^ employed an ANN algorithm to anticipate household electricity demand in Korea, demonstrating the efficacy of the prediction technique. Meanwhile, Liu et al.^66^ also used an artificial neural network to predict electricity consumption in China, demonstrating the model's outstanding precision. Khalil et al.^67^ developed an ANN approach to anticipate a building's cooling and heating requirements. The model addressed several variables including wall thickness, and orientation, glazing density distribution, glazing, roofing surface, surface area, relative compactness, and overall height. The dataset used for model construction was obtained from existing publications. Furthermore, Rahman et al.^68^ developed a recurrent neural network model to forecast medium- to long-term power demand patterns in both residential and commercial buildings, recognizing their significant effect on total electrical usage in the United States. Tartibu and Kabengele^69^ proposed an innovative way to estimate South Africa's prospective consumption of energy using an ANN. The proposed technique for estimating power consumption has been verified and evaluated using data from the Council for Scientific and Industrial Research spanning 2014 to 2050. The findings demonstrated that the ANN was capable of accurately predicting energy demand. Fayaz et al.^70^ presented a technique for evaluating short-term home energy use. This system had four separate layers: data gathering, preparation, forecasting, and validation. The research employed real data from four multi-story buildings in Seoul, South Korea, to demonstrate the efficiency of the proposed approach. Alshibani^7^ identified the factors impacting the schools’ energy consumption in hot and humid climate weather conditions. Also investigated the influence of the identified factors on the energy consumption of school facilities. Mohammed et al.^71^ proposed a regression model for predicting the energy consumption of schools in Saudi Arabia. The results revealed the regression models are proposing tools for estimating energy consumption.

The literature review indicated that there is a need to develop efficient models for predicting the energy consumption of educational buildings. Therefore, this paper proposes effective artificial intelligence approaches to estimate the energy consumption of educational buildings. Specifically, this paper proposes machine learning algorithms based on decision trees, K-nearest neighbors, gradient boosting, and long-term memory networks for estimating the energy consumption of school buildings. Real data are collected to develop and evaluate the proposed model. In addition, the variables that influence the energy consumption of educational buildings are determined based on the literature and experts. The relationship between the input factors and the energy consumption is investigated using the Pearson correlation test. The collected data are filtered and prepared. Moreover, the hyperparameters of the proposed models are tuned optimally.

## Material and method

This paper proposes machine learning algorithms for estimating the energy consumption of educational buildings. These models include decision trees, K-Nearest neighbor, gradient boosting, and long-term memory networks. The development process begins with identifying the factors influencing energy consumption, which are identified based on both literature reviews and experts. The actual data on energy consumption and relevant input parameters are then gathered and separated into distinct subsets for training, validation, and testing. The collected data are filtered and analyzed to develop robust machine-learning models.

**Figure 1:**
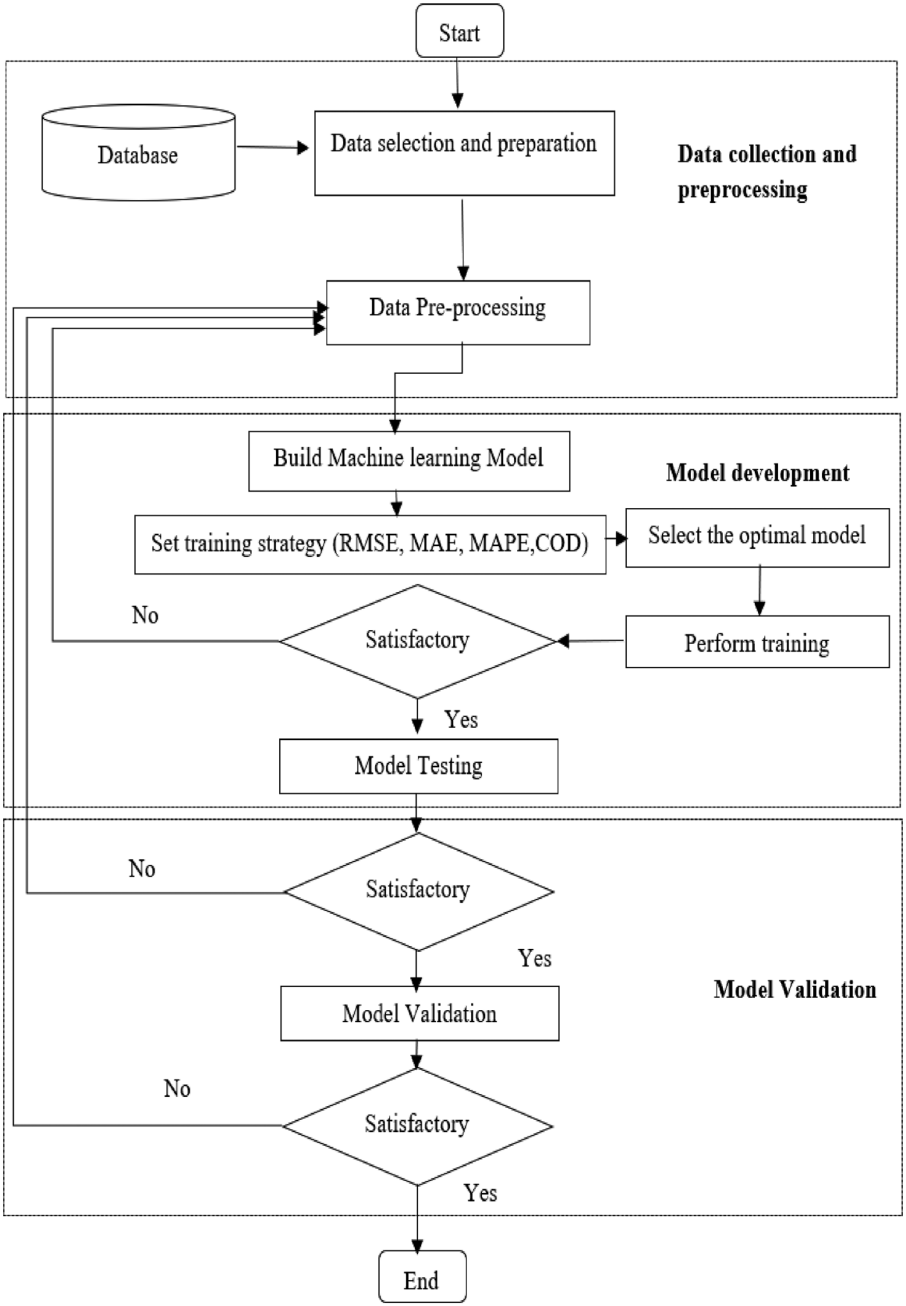
Overall flow diagram of the research method.

In this paper, descriptive statistics are conducted to provide concise summaries that facilitate understanding and interpretation of the collected data. A Scatter matrix also is constructed to investigate relationships among multiple variables within a dataset and to offer a comprehensive overview of how variables interact. The correlation between the input variables and the target, which is the annual energy consumption of educational buildings, is investigated. Correlation statistics are thoroughly applied to all input variables to identify redundant variables and explore the relationship between the factors and energy consumption. Moreover, parallel coordinate plots are developed to provide a comprehensive visual representation of data while also providing insights into the dataset's underlying structure. The second phase of the research methodology is to select and train the machine learning algorithm that will estimate the energy usage of educational buildings on a yearly bases. The optimal machine learning structure is selected so that the Coefficient of Determination (COD), Mean Absolute Percentage Error (MAPE), Mean Absolute Error (MAE), and Root Mean Square Error (RMSE) are minimized. The model's performance is then evaluated using the testing dataset. Figure 1 depicts the research methodology for the proposed model.

### Data collection

Direct data collection from educational buildings has replaced paper records as the most efficient way to monitor energy usage. This method seeks to shorten the amount of time needed to collect data. The data gathered covered a wide range of factors that affect energy use and was used as input for a machine-learning model. one parameter was the energy consumption, which was expressed in kWh annually and designated as the output. The dataset was established by filtering real energy consumption data from 352 educational facilities in order to eliminate any outliers. Eleven input variables and one output variable were selected from this refined dataset. In particular, some variables—like different kinds of lamps—were left out of the inputs since they had less of an effect on consumption than other variables. Table 1 illustrates the primary variables impacting the consumption of energy of educational buildings, which were employed as inputs for the machine learning models.

**Table 1:** Machine learning models input variables.

Input variables	Description
City	This factor describes the city in which the educational buildings are located. Nine different locations have been identified to collect data
Number of floors	The floors number of the educational buildings
Total built area	The sum of all enclosed spaces within educational buildings, including classrooms, offices, libraries, laboratories, and others
Total roof area	It refers to the one-floor area
Type of school	Four various educational types were investigated
Number of students	The sum of all individuals enrolling in an educational institution
Number of staff	It includes all the non-academic and academic workers
Age of building	It indicates how long it has been since its construction or completion
Number of classrooms	Total number of classes in the educational buildings
Total air-conditioned area	It refers to the total square footage or square meters of indoor space within a building or buildings that are cooled or heated by air conditioning systems
AC capacity	It refers to the total cooling or heating capacity used in air conditioning

### Data pre-processing

Data pre-processing plays a crucial role in the development of machine learning models. It involves a set of steps designed to prepare raw data for analysis. In this paper, descriptive statistics are used to better understand the interpretation of data science algorithms. The average, standard deviation, maximum, minimum, and quartile values for each parameter are calculated. In addition, different visualization tools to understand the behavior of the data are used. Data visualization is an effective tool for interpreting and communicating insights derived from complex datasets. By converting raw data into graphical representations, we can reveal the patterns, trends, and relationships that would otherwise be hidden. Through visualization, we can overcome the limitations of tabular data and gain a thorough understanding of its inherent structure and characteristics. This deeper understanding enables us to draw meaningful conclusions and make informed decisions. In this research, we also use the scatter matrix to perform a detailed pairwise comparison of variables, revealing their interrelationships and individual distributions. Moreover, we use a parallel coordinate plot to visualize multivariate data, allowing us to discern intricate patterns and correlations among the dataset's various dimensions, with a particular focus on annual energy consumption in schools. These visualization techniques not only illuminate complex data landscapes but also help to identify key insights that are critical for informed decision-making and strategic planning. Furthermore, the relationship between the input factors and between the inputs and output is investigated. Pearson's coefficient of correlation is used to improve understanding of the relationship between the input variables and the target, which is the annual energy consumption of educational buildings.

### Complex artificial intelligence models through machine and deep learning

In this paper, various machine-learning models are proposed to estimate the energy consumption of educational buildings. These techniques include decision trees, K-nearest neighbors, gradient boosting, and long short-term memory. A brief description of each algorithm is provided in the subsequent subsections:

1. Decision Tree: A decision tree algorithm is a machine learning technique that partitions input data recursively based on feature values, resulting in the prediction of a target variable. Starting with the entire dataset at the root node, the algorithm selects the best attribute to split the data into subsets, to maximize information gain, minimize impurity, or reduce variance, depending on the task. This process continues iteratively, resulting in a tree-like structure with internal nodes representing decision points based on feature values and leaf nodes representing predicted outcomes. Overfitting can be prevented by using a variety of stopping criteria, such as maximum depth or minimum samples per leaf. Pruning techniques can help refine the tree by removing unnecessary branches. Prediction involves traversing the tree from the root to a leaf node based on the attribute values of a new instance, and the majority class or average value in the leaf node is assigned as the prediction^72^.
2. K-Nearest Neighbor: The k-nearest neighbors is a useful and simple technique for prediction and classification. It works by identifying the k closest data points (neighbors) in the feature space to a given query instance and then making predictions based on their labels or values. In classification, the predicted class is usually determined by a majority vote of the k nearest neighbors, whereas in regression, the predicted value is frequently the average of the k nearest neighbors' values. The number of neighbors to consider (k) is an important hyperparameter that can have a significant impact on the algorithm's performance. A lower value of k results in more flexible decision boundaries but may increase sensitivity to noise, whereas a higher value of k smooths out the decision boundaries but may oversimplify the model. The k-NN algorithm is a popular choice for a variety of machine learning tasks due to its simplicity and effectiveness, particularly when the underlying data distribution is unknown or interpretability is required^73^.
3. Gradient Boosting: Gradient boosting is an effective machine-learning technique for both regression and classification tasks. It constructs a predictive model sequentially using an ensemble of weak learners, typically decision trees. The algorithm works by fitting a series of trees to the previous trees' residuals (or gradients), with each new tree attempting to correct the errors made by the predecessors. Gradient boosting, an iterative process, builds a strong learner by combining the predictions of multiple weak learners, frequently achieving high predictive accuracy. Gradient boosting's key components include the selection of a loss function, the learning rate, which controls the contribution of each tree to the ensemble, and the maximum depth or complexity of the individual trees^74^.
4. Long Short-Term Memory: Long Short-Term Memory (LSTM) is a recurrent neural network structure that aims to overcome the limitations of traditional RNNs in recording extensive dependents within data that is ordered. LSTMs use a gating mechanism consisting of input, forget, and output gates to control information flow throughout the network, in contrast to traditional RNNs, which have difficulties with the vanishing gradient problem when learning from remote dependencies. With the use of this gating mechanism, long sequences of data can be retained with greater relevance while lowering the likelihood of signal degradation. This allows LSTMs to selectively update and discard information over time. Furthermore, LSTMs include a cell state that acts as a conveyor belt, carrying information across time steps and facilitating the flow of gradients during training. As a result, LSTMs have become a key component in sequence modeling tasks, outperforming traditional RNN architectures^75^.

### Performance evaluation of machine learning models

Different assessment measures are utilized to assess the accuracy of the proposed machine learning algorithms. These measures include Root Mean Square Error (RMSE), Mean Absolute Error (MAE), Mean Absolute Percentage Error (MAPE), and Coefficient of Determination (COD).

1. Mean Absolute Error^76^: It measures the mean absolute difference between the values that a model predicts and the values that are actually in a dataset. It is given by the following formula:

$$ {\text{MAE}} = \frac{1}{n}\sum\limits_{i = 1}^{n} {\left| {y_{i} - \hat{y}_{i} } \right|} $$

where the yi is the actual value of the energy consumption at point , n represents the number of educational buildings, and $\widehat{{y}_{i}}$ is predicted value of the energy consumption at point.

2. Root Mean Square Error^77^: It calculates the square root of the average squared variances between the actual and anticipated values in a dataset. Mathematically, RMSE is calculated as:

$$ RMSE = \sqrt {\frac{1}{n}\sum\limits_{i = 1}^{n} {(y_{i} - \hat{y})^{2} } } $$

3. Mean Absolute Percentage Error^78^: It calculates the mean absolute percentage difference between a dataset's actual values and its predicted values. Mathematically, MAPE is calculated as:

$$ {\text{MAPE}} = \frac{1}{n}\sum\limits_{i = 1}^{n} {\frac{{\left| {y_{i} - \hat{y}_{i} } \right|}}{{y_{i} }}} \times 100\% $$

4. Coefficient of Determination^79^: It demonstrates the percentage of a machine learning model's dependent variable's variance that can be predicted based on the independent variables. In other words, COD quantifies the goodness of fit of the model to the actual data. It is given by the following formula:

$$ COD = 1 - \frac{{\sum\nolimits_{i = 1}^{n} {(y_{i} - \hat{y}_{i} )^{2} } }}{{\sum\nolimits_{i = 1}^{n} {(y_{i} - \overline{y})^{2} } }} $$

## Results and discussion

### Descriptive statistics of the data variables

The descriptive statistics is fundamental to understand the interpretation of data science algorithms, which is depicted in Table 2. The study encompassed data collection from 352 schools, presenting a comprehensive analysis of various structural and operational characteristics. The mean number of floors was approximately 3.26, with the built area averaging at 2,596.59 square meters and the roof area at 4,165.63 square meters. School types varied with a mean value suggesting a mix of primary and secondary education facilities. On average, schools had 381.10 students and 28.18 staff members. The average age of the school buildings was noted to be approximately 32.82 years, reflecting a moderate level of infrastructural maturity. Classrooms averaged at 14.26 per school, indicating moderate to large school sizes. Air-conditioned areas averaged 817.71 square meters, with the AC capacity averaging at 160.12 tons of refrigeration. The mean annual energy consumption of the schools was a substantial 410,968 kWh/year. The data displayed a standard deviation in total built area and roof area of 1902.62 and 649.17 square meters, respectively, indicating significant variability among the sizes of the schools surveyed. The standard deviation in the number of students and staff was 217.57 and 9.35, respectively, which illustrates a diverse range of school populations and staffing levels. The variability in air-conditioned area and AC capacity (std of 354.60 m^2^ and 55.82 tons of refrigeration) reflects different approaches to climate control.

**Table 2:** Description statistics of the input variables and the target variable.

	City	Number of floors	Total built area	Total roof area	Type of school	Number of students	Number of staff	Age of building	Number of classrooms	Total air-conditioned area	AC Capacity	Annual consumption
Count	352	352	352	352	352	352	352	352	352	352	352	352
Mean	3.255682	2.596591	4165.628	1524.457	3.289773	381.1023	28.17614	32.82386	14.25852	817.7088	160.1151	410,968
Std	2.214295	0.591285	1902.623	649.1695	0.912782	217.5674	13.01096	9.353318	6.121624	354.6029	55.81558	143,405.3
Min	1	1	360.63	360.63	1	10	The	11	2	117	38	99,274.95
25%	1	2	2622	1178	3	229	19	24	10	585	134	342,312.7
50%	3	3	4275	1425	4	366	28	35	13	760.5	172	470,037.6
75%	5	3	5940	1980	4	494.5	37	39	18	1053	200	481,798.8
Max	9	3	6900	3078	4	1259	76	58	40	2340	250	683,191.8

### Data visualization and interpretation

**Figure 2:**
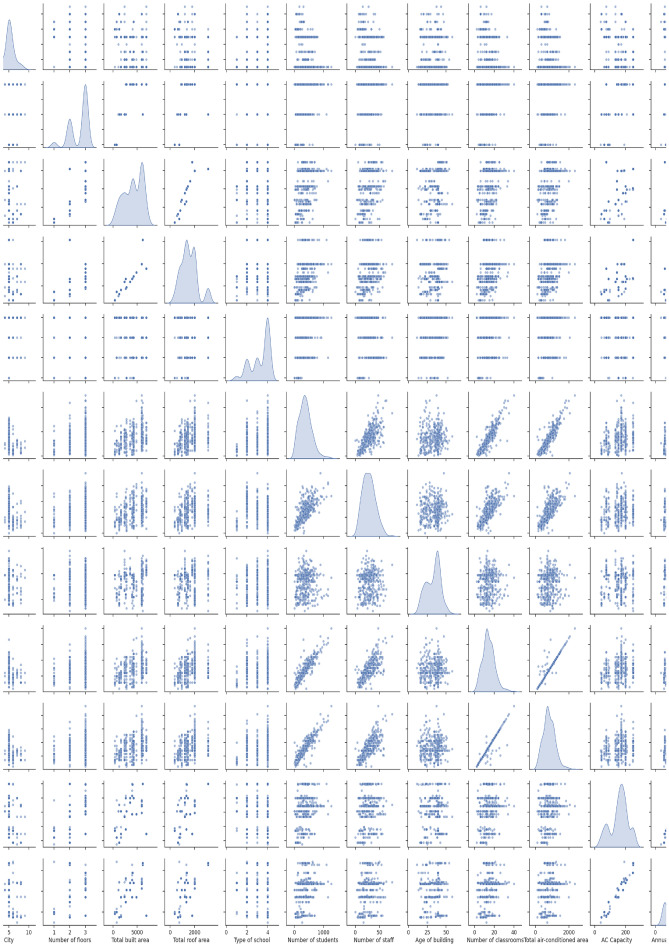
Scatter matrix of the variables. A full resolution figure can be reviewed on the link: https://doi.org/10.6084/m9.figshare.26024836.

The data visualization is supported through the usage of a scatter matrix which is displayed in Fig. 2. The scatter matrix provided allows for a comprehensive pairwise comparison of the variables in relation to one another, including their individual distributions. This form of analysis is instrumental in discerning the nature of relationships across multiple dimensions of the dataset, especially focusing on how they may correlate with annual energy consumption in schools. Analyzing the histograms on the diagonal, a distribution of each variable can be noted. For instance, 'City' appears to have a uniform distribution, whereas 'Annual Consumption' seems to be skewed, with a high occurrence of lower values and fewer instances of high energy consumption, suggesting a concentration of schools with lower energy use. The scatter plots off the diagonal offer insights into bivariate relationships. For example, 'Total Built Area' vs. 'Total Roof Area' displays a linear trend, indicating a strong positive correlation, as expected since larger built areas typically result in larger roof areas. Similarly, 'Number of Students' vs. 'Number of Classrooms' shows a clear positive trend, highlighting that schools with more students tend to have more classrooms. Considering 'Annual Consumption', the plots against variables like 'Total Built Area', 'Total Roof Area', and 'AC Capacity' reveal positive correlations, as indicated by the concentration of points along an upward trajectory. This supports the hypothesis that larger school sizes and greater AC capacities are associated with higher energy consumption. Conversely, variables such as 'Type of School' show a more dispersed scatter with 'Annual Consumption', suggesting a less direct or weaker correlation. This implies that the type of school may not be a strong determinant of its energy consumption levels. The 'Number of Floors' does not show a distinct trend in relation to 'Annual Consumption', which might indicate that the number of floors in a school is not a straightforward predictor of its energy use, possibly due to variations in building design and usage patterns. It is also interesting to note the patterns in 'Number of Students', 'Number of Staff', and 'Number of Classrooms', which when plotted against 'Annual Consumption', show a more cloud-like distribution with a slight positive correlation. This suggests that while larger numbers in these categories can lead to increased energy use, the relationship is not as pronounced or linear as with physical building characteristics. Finally, the relationship between 'Age of Building' and 'Annual Consumption' does not present a clear pattern, indicating that the age may not have a straightforward impact on energy usage. This could be due to a variety of factors, such as renovations, maintenance practices, or the installation of energy-efficient systems in older buildings. In summary, the scatter matrix provides a rich visual framework for understanding the complex interrelationships between school characteristics and energy consumption. It highlights significant variables that warrant closer examination and could inform strategies for energy efficiency, facility design, and management within the educational sector.

**Figure 3:**
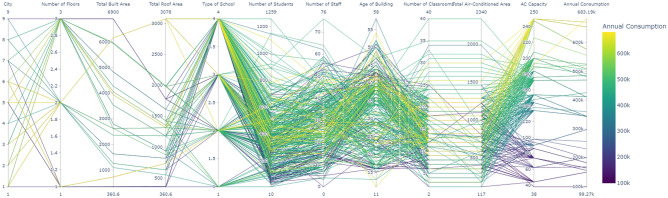
Parallel coordinate plot of the variables. A full resolution figure can be reviewed on the link: https://doi.org/10.6084/m9.figshare.26025634.

The visualization of data is higher order dimensions is quite tricky, therefore a parallel coordinate plot of all the variables is displayed in Fig. 3, whereas, the line colors on it are only tracing the variability in the annual energy consumption. Therefore, the parallel coordinate plot provides a visual representation of multivariate data, allowing us to discern patterns and relationships between the various dimensions of the dataset, particularly in relation to annual energy consumption in schools. From the visualization, it is evident that certain variables exhibit a greater range of values and are more densely interwoven with annual consumption, which is color-coded from purple to yellow, indicating low to high energy usage, respectively. Notably, the lines connecting 'Total Roof Area', 'Number of Students', 'Number of Classrooms', and 'AC Capacity' to 'Annual Consumption' display a gradient trend, transitioning from cooler to warmer colors as the values increase. This suggests a positive correlation where higher values in these variables tend to be associated with greater energy consumption. The variables 'City' and 'Number of Floors', while also connected to varying degrees of energy consumption, show less of a clear gradient, indicating that the relationship with energy use may be influenced by other factors or is less directly correlated. Furthermore, the 'Type of School' and 'Age of Building' appear to have a more diffuse distribution of colors across their range, implying a weaker or less direct relationship with energy consumption. These lines do not follow a clear pattern, suggesting that the type of school and the age of the building are not as strongly predictive of energy usage as the size or occupancy-related variables. It is also noteworthy that 'Total Built Area', much like 'Total Roof Area', displays a concentration of warmer colors at higher values, supporting the idea that larger physical dimensions are indicative of increased energy needs. The densely packed lines connecting 'Number of Classrooms' and 'Total Air-Conditioned Area' with higher annual consumption rates further underscore the significant impact of space usage on energy consumption. The correlation appears stronger as the air-conditioned space is a direct consumer of energy, primarily through cooling systems. In contrast, the visual representation does not suggest a strong direct relationship between 'Number of Staff' and energy consumption, as evidenced by the more uniformly distributed color range across its values. Lastly, 'AC Capacity' shows a very strong association with energy consumption, indicated by a predominance of yellow lines as AC capacity increases, reinforcing the assumption that air conditioning is a substantial component of a school's energy profile. Overall, the parallel coordinate plot underlines the multidimensional nature of energy consumption in educational facilities. It highlights the significance of spatial parameters and student density as strong indicators of energy use, while also illustrating the intricate relationships and potential confounding factors that must be considered in the pursuit of energy efficiency and sustainability in school design and operation.

**Figure 4:**
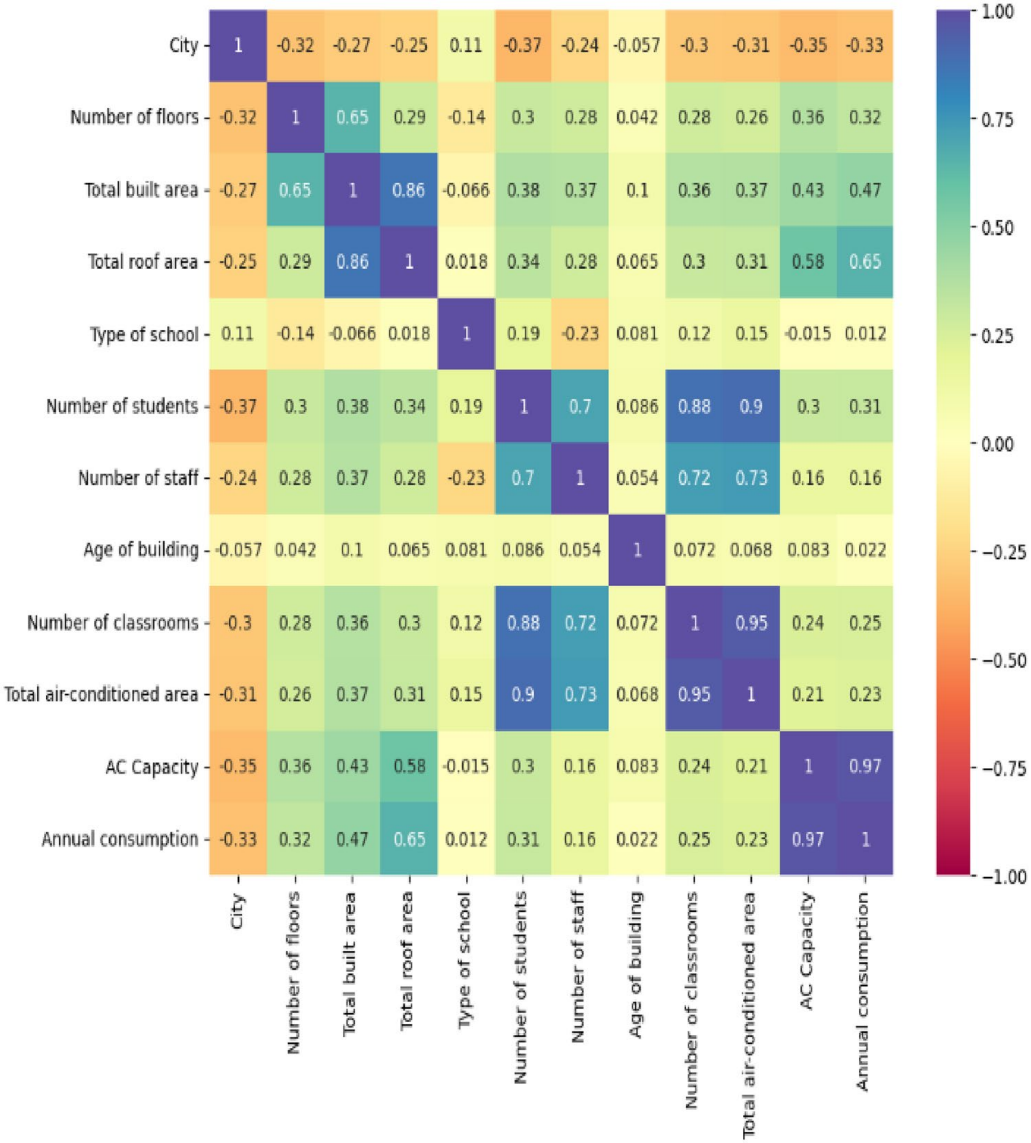
Heat map based upon Pearson coefficient of correlation.

The coefficient of correlation based upon the mathematics of Pearson can improve the understanding between the correlation of the input variables and the target which is annual energy consumption of the educational buildings, which is depicted in Fig. 4. Upon analyzing the Pearson Coefficient of Correlation between various school characteristics and their annual energy consumption, several interesting relationships emerge. The city shows a moderate negative correlation (− 0.3256) with energy consumption, indicating that certain urban factors might be leading to more efficient energy use or that different locales may have varying energy requirements. In contrast, the number of floors within a school displays a positive correlation (0.3218) with energy consumption. This could be due to the additional energy required for heating or cooling larger vertical spaces and the use of elevators. The total built area and roof area of schools show positive correlations of 0.4678 and 0.6511 with annual energy consumption, respectively, which are among the strongest observed in the dataset. These correlations suggest that as the physical footprint of a school increases, so does its energy demand, likely due to the larger volumes of space requiring climate control. Interestingly, the type of school has an almost negligible positive correlation (0.0119) with energy consumption. The student and staff numbers show positive correlations with energy consumption, 0.3088 and 0.1589 respectively, though the strength of these relationships is varied. The number of students has a stronger correlation, which could be due to the fact that more students likely require more resources, including lighting, heating, cooling, and technology, all of which contribute to energy use. The number of staff has a weaker positive correlation, indicating that staff numbers alone are not as significant a determinant of energy consumption as student numbers. Age of the building shows a very weak positive correlation (0.0217) with energy consumption, suggesting that older buildings may not necessarily consume more energy than newer ones, possibly due to factors such as construction materials, design, or retrofits. The number of classrooms and the total air-conditioned area both show weak to moderate positive correlations (0.2495 and 0.2301, respectively) with energy consumption. More classrooms could imply more space to heat, cool, and light, whereas more air-conditioned space directly relates to higher energy use due to the demands of cooling systems. Finally, the AC capacity shows a very strong positive correlation (0.9703) with annual energy consumption. This is to be expected, as air conditioning is a major energy consumer within buildings, especially in climates that require it year-round or for large parts of the year. In summary, the size of the school (in terms of both physical dimensions and occupancy) and the capacity of air conditioning systems are prominent factors in energy consumption. The relationship between these variables and energy use is multifaceted, with implications for energy management, sustainability initiatives, and the design of educational facilities. While certain correlations are stronger than others, each variable contributes to the overall energy footprint of a school, and understanding these relationships is key for developing strategies to reduce energy consumption and promote efficiency.

### Artificial intelligence models to predict the energy consumption in educational buildings

Various artificial intelligence methods from the machine learning block like decision tree, K-Nearest Neighbors, Gradient Boosting, and one deep learning method which is Long-Short-Term Memory was implemented, and the construction of the hyper-parameters are developed as follows:

1. **Decision tree:** In this study, a Decision Tree Regressor algorithm was developed to model and predict annual energy consumption in schools, considering various structural and operational characteristics as predictors. The model was constructed using a robust machine learning pipeline employing Scikit-learn, a popular Python library for data science and machine learning. The predictive features (X) incorporated in the model were: 'Number of floors', 'Total built area', 'Total roof area', 'Number of students', 'Number of staff', 'Age of building', 'Number of classrooms', 'Total air-conditioned area', and 'AC Capacity', with the target variable (y) being 'Annual consumption'. The dataset was divided into a training set (80%) and a testing set (20%), ensuring the model's ability to generalize to new, unseen data. To optimize the model's performance, a grid search with five-fold cross-validation was executed over a predefined range of hyperparameters. The parameters included various depths, minimum samples for splitting and at leaf nodes, maximum features at each split, and the maximum number of leaf nodes. The grid search aimed to find the optimal combination of parameters that minimized the negative mean squared error (MSE). The best hyperparameters for the Decision Tree were determined to be a maximum depth of 5, utilizing all features at each split ('auto'), a limit of 10 maximum leaf nodes, a minimum of 10 samples at each leaf node, and a minimum of 20 samples required to split a node. This configuration indicates a model complex enough to capture underlying patterns, yet restrained to prevent overfitting.
2. **K-Nearest Neighbor:** A K-Nearest Neighbors (KNN) Regressor algorithm was implemented to forecast the annual energy consumption in educational institutions, leveraging a variety of structural and operational features as predictive variables. The model was constructed using Scikit-learn, an esteemed Python library renowned for its proficiency in machine learning and data science endeavors. The feature set incorporated into the model constituted all available variables except for the 'Annual consumption', which served as the target variable (y). The dataset was prudently partitioned into training and testing subsets, with an 80–20 split, reinforcing the model's robustness in generalizing to unseen data points. To ascertain the most efficacious configuration of hyperparameters, an exhaustive grid search, coupled with five-fold cross-validation, was executed. This entailed a methodical exploration of different numbers of neighbors, weight schemes, algorithms, and distance metrics. The grid search's chief objective was to pinpoint the hyperparameter combination that would minimize the negative mean squared error, thereby enhancing the model's predictive accuracy. The implementation of the K-Nearest Neighbors (KNN) algorithm involved an extensive hyperparameter tuning process using GridSearchCV, a method that systematically works through multiple combinations of parameter tunes, cross-validating as it goes to determine which tune gives the best performance. The algorithm evaluated a total of 64 candidate models across 5 different folds of the dataset, resulting in 320 individual fits. Subsequent predictions on both the training and testing data were then generated using this refined model.
3. **Gradient Boosting:** Gradient Boosting Regressor model was implemented to forecast annual energy consumption in schools based on a set of selected features. Employing Scikit-learn's comprehensive toolkit, the integrated features that represent both physical characteristics and demographic factors of schools, such as 'Number of floors', 'Total built area', 'Total roof area', 'Number of students', 'Number of staff', 'Age of building', 'Number of classrooms', 'Total air-conditioned area', and 'AC Capacity', with the target variable being the 'Annual consumption'. A systematic division of the dataset facilitated the creation of distinct training and testing cohorts, comprising 80% and 20% of the data, respectively. This was followed by standardization of the features to normalize the data, enabling more effective learning by the model. The model's calibration was meticulously conducted through a GridSearchCV process that spanned a diverse array of hyperparameters, including the number of estimators, learning rate, subsample rate, and tree depth, across fivefold cross-validation to enhance the reliability of the results. The objective was to discover the optimal settings that minimize the negative mean squared error, a metric that captures the average squared difference between the estimated values and the actual value. The search across the hyperparameter space evaluated 54 unique candidates in 270 fitting iterations, rigorously assessing each combination to ensure the selection of the most predictive model configuration.
4. **Long-Short Term Memory (LSTM)**: LSTM neural network model was developed to predict the annual energy consumption of schools. This model represents a sophisticated form of recurrent neural network capable of learning order dependence in sequence prediction problems. Normalization of the data was paramount, achieved through the application of *MinMaxScaler*, which scaled the feature set and target variables to a bounded interval of [0, 1]. This scaling facilitated the neural network's convergence during training by providing numerical stability and improved efficiency. The dataset was then divided into a training subset accounting for 80% of the data and a testing subset forming the remaining 20%, with the intention of validating the model's predictive performance on unseen data. The LSTM model's architecture was constructed with an input layer designed to accept the reshaped feature set, followed by an LSTM layer with 50 units and 'relu' activation, and culminating in a Dense output layer for prediction. The model's learning process involved an 'adam' optimizer and mean squared error loss function across 50 epochs, with a batch size of 32, underscoring the iterative refinement of the model's weights.

**Figure 5:**
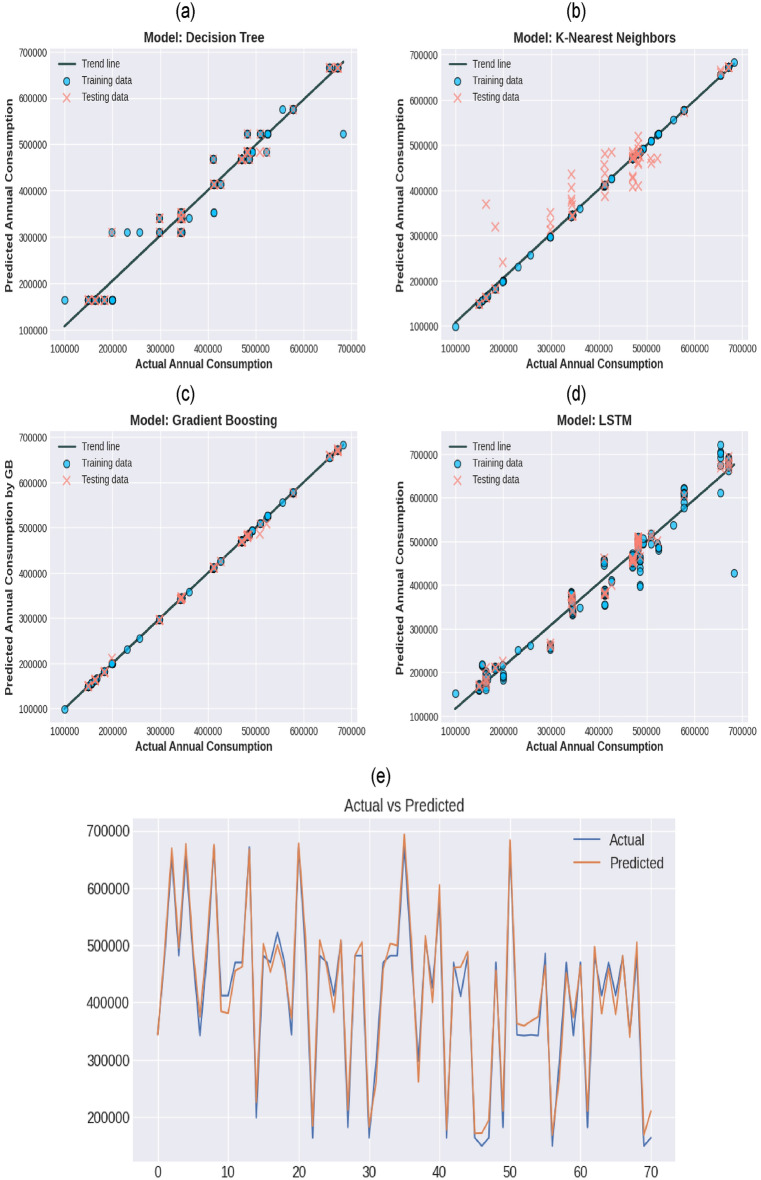
Regression fit of (**a**) Decision trees, (**b**) K-nearest neighbor, (**c**) gradient boosting, (**d**) LSTM. (**e**) LSTM representation in time series.

Once the computer program for the corresponding machine learning algorithm is developed, afterwards, it is evaluated graphically and quantitatively. The graphical evaluation can be noted in Fig. 5 which shows the regression for all of the techniques. Figure 5 comprises four panels labeled (a), (b), (c), and (d), each representing a scatter plot comparing actual versus predicted annual energy consumption using different predictive models: Decision Tree, K-Nearest Neighbors (KNN), Gradient Boosting, and Long Short-Term Memory (LSTM) neural network, respectively. The actual annual consumption is plotted along the x-axis, while the predicted values are on the y-axis for each model. The presence of a trend line in each graph provides a reference for perfect prediction, where the predicted values would ideally match the actual consumption exactly. The points are color-coded, with blue dots representing training data and red crosses denoting testing data. This color scheme allows for immediate visual discrimination between the model's performance on the data it was trained on and its predictive power on new, unseen data. In the similar context, Fig. 5e explains another way of presenting LSTM data points.

The Decision Tree model displays a reasonable spread of training data around the trend line but shows some variance in the predictions of testing data, as seen by the red crosses that stray further from the trend line. This might suggest a degree of overfitting to the training data, or it could simply reflect the model's inherent variance when dealing with more complex patterns not captured during training. In contrast, the KNN model exhibits a more consistent pattern of prediction across both training and testing datasets, with the majority of points closely aligned with the trend line. However, there are noticeable outliers in the testing data, which may imply that while the model generalizes well in most cases, it may be sensitive to certain data points or to noise within the dataset. The Gradient Boosting scatter plot demonstrates an impressive adherence of the data points to the trend line, with both training and testing data showing tight clustering around this line. This indicates that the model not only learned the training data effectively but also generalized well to the testing data, showcasing the strength of Gradient Boosting in capturing complex relationships within the data. Lastly, the LSTM neural network, known for its ability to capture sequential and temporal relationships, shows a distinctive pattern. Training data is well-fitted, and testing data, while slightly more scattered, still follows the trend line reasonably well. Notably, the LSTM shows more variation in the higher range of actual consumption values, which could indicate the model's different handling of more extreme values or its response to the nuances of the sequence-based data. When comparing the four models, Gradient Boosting (Panel c) seems to outperform the others in terms of the tightness of data points around the trend line, suggesting a high accuracy and good generalization. The KNN and LSTM model also generalizes well but with some noted exceptions.

Each model's unique characteristics influence its performance, with tree-based models like Decision Trees and Gradient Boosting often handling non-linear relationships effectively, while KNN relies on localized data patterns, and LSTM models excel in capturing temporal dynamics. The selection of the best model would therefore depend on the specific characteristics of the dataset and the performance metric of interest, whether it be accuracy, robustness, or the ability to generalize from limited data.

Our analysis compares four machine learning techniques across two phases: training and testing. Table 3 provides performance metrics such as Root Mean Square Error (RMSE), Mean Absolute Error (MAE), Mean Absolute Percentage Error (MAPE), and Coefficient of Determination (COD). The Decision Tree model exhibits strong performance on the training data, with a relatively low RMSE of 20,716.25 and MAE of 10,764.99, indicating good fit. The MAPE value of 3.580435 shows that, on average, the prediction error is about 3.58% of the actual value, which is respectable in practical applications. The high COD of 0.978928 for training and 0.980852 for testing indicates that the model explains a large portion of the variance in the data, confirming its effectiveness. The K-Nearest Neighbors model has significantly higher errors, with RMSE on training data as high as 38,429.4, which may be indicative of overfitting, considering the perfect COD of 0.934134. The testing phase does not fare much better, with a further increased RMSE of 40,603.04. This could be due to the model's sensitivity to the local structure of the training data, which doesn't generalize well. In contrast, Gradient Boosting outperforms other models during the training phase with a remarkably low RMSE of 374.7943 and an almost perfect COD, signifying that the model can almost perfectly predict the variations within the training dataset. However, the model's ability to generalize is not as clear without the testing phase COD, even though the testing RMSE increases to 3559.443. The LSTM network, designed to capture long-term dependencies and temporal dynamics in sequential data, shows good performance on both training and testing data. The RMSE and MAE values are moderate, with the model achieving a higher COD value on the testing set (0.975606) than on the training set (0.956432), which suggests it generalizes well and confirms its suitability for time-series forecasting like annual consumption. Our dataset's characteristics, with a substantial standard deviation, imply significant variability in annual consumption, which is a common challenge in time-series prediction. The performance metrics suggest that some models manage this variability better than others, with Gradient Boosting and LSTM standing out in terms of their ability to handle diverse data ranges, from the minimum consumption of approximately 99,274.95 to the maximum of 683,191.8. This evaluation demonstrates the importance of choosing the right model for time-series forecasting. While Decision Trees and KNN provided baseline performances, the Gradient Boosting and LSTM models showed advanced capabilities, which could be attributed to their sophisticated handling of non-linear relationships and temporal dependencies in the data.

**Table 3:** Evaluation of machine learning models.

Technique	Phase	RMSE	MAE	MAPE	COD
Decision tree	Training	20,716.25	10,764.99	3.580435	0.978928
	Testing	20,082.19	10,344.31	3.457668	0.980852
KNN	Training	384,294.9	20,135.64	5.874564	0.934134
	Testing	40,603.04	21,174.79	6.965188	0.921724
Gradient boosting	Training	374.7943	236.8721	0.064815	0.999993
	Testing	3559.443	1510.667	0.413988	0.999398
LSTM	Training	29,787.81	21,934.77	6.538339	0.956432
	Testing	22,666.59	19,915.98	6.294538	0.975606

## Conclusion

Educational facilities, including schools, represent a major piece of infrastructure that significantly impacts economic, social and environment sustainable development. They contribute to approximately 37% of the carbon dioxide emissions associated with energy use and procedures. This paper presents an investigation study for prediction energy consumption of school buildings utilizing machine learning technologies. The methodology followed to meet the objectives of the study consists of four main steps. The first step begins with identifying the variable impacting consumption of energy in school buildings based on both review of the literature and meeting the experts. In the second step, the actual data on energy consumption was collected, filtered, and analyzed to develop robust machine-learning models. The third step comprises of separating the collected data into distinct subsets for training, validation, and testing. The fourth step involves the development and the investigation of several artificial intelligence-based models for energy consumption of educational buildings including decision trees, K-nearest neighbors, gradient boosting, and long-term memory networks.

The study revealed that the decision tree-based prediction model illustrates strong performance with an average prediction error of about 3.58%. The K-Nearest Neighbors model has significantly higher errors, with RMSE on training data as high as 38,429.4. Conversely, Gradient Boosting perfectly predicts energy consumption of school buildings. The performance metrics suggest that some models manage this variability better than others. Gradient boosting and LSTM stand out in terms of their ability to handle diverse data ranges, from the minimum consumption of approximately 99,274.95 to the maximum of 683,191.8. Furthermore, the relationship between the input factors and the annual consumption of energy of educational buildings illustrates that school sizes and AC capacities are the most impacted variable associated with higher energy consumption. While 'Type of School' is less direct or weaker correlation with 'Annual Consumption'.

It is essential to note that this study was carried out based on typical school facilities in hot climate. Thus, the results of this research may not be suitable to other school types and/or climates. Moreover, extended study can be conducted in the future to include different school types, structure system, and different climate.

Future research should focus on enhancing the role of educational buildings as interactive learning environments that promote sustainability principles. By using advanced AI-driven tools to optimize energy consumption, future studies could develop and implement smart, adaptive systems that not only manage energy use more efficiently but also serve as educational tools themselves. These systems could provide real-time data and simulations that allow students to observe, interact with, and learn from the building’s energy dynamics. This approach would not only improve the sustainability of educational facilities but also actively engage students with practical lessons on energy efficiency and environmental concepts, thereby embedding these critical concepts into their daily experiences and learning environments, as a part of lifelong learning.

## Data Availability

Data will be made available on request. Please request Professor Rasikh Tariq for the data simulation and Professor Awsan Mohammed for the experimentation.

## References

[1] Alfaoyzan FA, Almasri RA (2023). Benchmarking of energy consumption in higher education buildings in Saudi Arabia to be sustainable: Sulaiman Al-Rajhi University case. Energies.

[2] IEA. Buildings. Paris. License: IEA. https://www.iea.org/reports/buildings. CC BY 4.0 (2022).

[3] Zhao HX, Magoules F (2012). A review on the prediction of building energy consumption. Renew. Sustain. Energy Rev..

[4] Gassar AAA, Cha SH (2020). Energy prediction techniques for large-scale buildings towards a sustainable built environment: A review. Energy Build..

[5] Asimakopoulos DN, Doulamis AD (2019). Predictive analytics for energy consumption in educational buildings: A review of modeling techniques. Sustain. Cities Soc..

[6] Debnath KB, Mourshed M (2018). Forecasting methods in energy planning models. Renew. Sustain. Energy Rev..

[7] Alshibani A (2020). Prediction of the energy consumption of school buildings. Appl. Sci..

[8] Kim S-G, Jung J-Y, Sim MK (2019). A two-step approach to solar power generation prediction based on weather data using machine learning. Sustainability.

[9] Blumsack S, Fernandez A (2012). Ready or not, here comes the smart grid!. Energy.

[10] Zhong H, Wang J, Jia H, Mu Y, Lv S (2019). Vector field-based support vector regression for building energy consumption prediction. Appl. Energy.

[11] Zhao Y, Zhang C, Zhang Y, Wang Z, Li J (2020). A review of data mining technologies in building energy systems: Load prediction, pattern identification, fault detection and diagnosis. Energy Built. Environ..

[12] Wang J, Hou J, Chen J, Fu Q, Huang G (2021). Data mining approach for improving the optimal control of HVAC systems: An event-driven strategy. J. Build Eng..

[13] Darwazeh D, Duquette J, Gunay B, Wilton I, Shillinglaw S (2022). Review of peak load management strategies in commercial buildings. Sustain. Cities Soc..

[14] Jin W, Fu Q, Chen J, Wang Y, Liu L, Lu Y (2023). A novel building energy consumption prediction method using deep reinforcement learning with consideration of fluctuation points. J. Build. Eng..

[15] Gellert A, Fiore U, Florea A, Chis R, Palmieri F (2022). Forecasting electricity consumption and production in smart homes through statistical methods. Sustain. Cities Soc..

[16] Zhang W, Chen Q, Yan J, Zhang S, Xu J (2021). A novel asynchronous deep reinforcement learning model with adaptive early forecasting method and reward incentive mechanism for short-term load forecasting. Energy.

[17] Purnell K, Sinclair M, Gralton A (2004). Sustainable schools: Making energy efficiency a lifestyle priority. Aust. J. Environ. Educ..

[18] Rogora, A., & Dessì, V. Recent Examples of Low Energy and Sustainable Schools in Italy. In *22nd International Conference, PLEA 2005: Passive and Low Energy Architecture - Environmental Sustainability: The Challenge of Awareness in Developing Societies, Proceedings*, 1, 275–280. https://re.public.polimi.it/bitstream/11311/693887/1/plea%202005-%20esempi%20di%20scuole.pdf (2005).

[19] Zeiler W. & De Waard M. Some dutch examples of sustainable school concepts towards plus energy schools. In *28th Conference PLEA, Opportunities, Limits & Needs Towards an environmentally responsible architecture*. Lima: Pontificia Universidad Católica del Perú (2012).

[20] Ramírez-Montoya, M.S., Basabe, E., Carlos Arroyo, M., Patiño Zúñiga, I.A., & Portuguez Castro, M. Modelo abierto de pensamiento complejo para el futuro de la educación. Octaedro. https://hdl.handle.net/11285/652033 (2024).

[21] Passa J, Rompf D (2007). Energy efficient sustainable schools in Canada South. J. Green Build..

[22] Golshan M, Thoen H, Zeiler W (2018). Dutch sustainable schools towards energy positive. J. Build. Eng..

[23] Zhang, Q., Koh, B. B., & Ahn, Y. H. Energy saving technologies and sustainable strategies of sustainable school buildings: A case study of isaac dickson elementary school. *Int. J. Sustain. Build. Technol. Urban Dev.* **11**(2), 94–111. 10.22712/susb.20200008 (2020).

[24] Boeri A, Longo D (2013). Environmental quality and energy efficiency: sustainable school buildings design strategies. Int. J. Sustain. Dev. Plan..

[25] Msaddek MH, Moumni Y, Ayari A, El May M, Chenini I (2022). Artificial intelligence modelling framework for mapping groundwater vulnerability of fractured aquifer. Geocarto Int..

[26] Tao H, Al-Khafaji ZS, Qi C, Zounemat-Kermani M, Kisi O, Tiyasha T, Chau K-W, Nourani V, Melesse AM, Elhakeem M, Farooque AA, Pouyan NA, Khedher KM, Alawi OA, Deo RC, Shahid S, Singh VP, Yaseen ZM (2021). Artificial intelligence models for suspended river sediment prediction: state-of-the art, modeling framework appraisal, and proposed future research directions. Eng. Appl. Comput. Fluid Mech..

[27] Baki S, Koutiva I, Makropoulos C (2012). A hybrid artificial intelligence modelling framework for the simulation of the complete, socio-technical, urban water system. Eng. Appl. Comput. Fluid Mech..

[28] Méndez-Suárez M, García-Fernández F, Gallardo F (2019). Artificial intelligence modelling framework for financial automated advising in the copper market. J. Open Innov. Technol. Mark. Complex..

[29] Reddy, R. S., Keesara, N., Pudi, V., & Garg, V. Plug load identification in educational buildings using machine learning algorithms. In *Proceedings of BS2015: 14th conference of international building performance simulation association, Hyderabad, India*, pp. 1940–1946 (2015).

[30] López-Pérez LA, Flores-Prieto JJ (2023). Adaptive thermal comfort approach to save energy in tropical climate educational building by artificial intelligence. Energy.

[31] Hosseini P, Nikbakht Naserabad S, Keshavarzzadeh AH, Ansari N (2022). Artificial intelligence-based tri-objective optimization of different demand load patterns on the optimal sizing of a smart educational buildings. Int. J. Energy Res..

[32] Lee MJ, Zhang R (2024). Human-centric artificial intelligence of things-based indoor environment quality modeling framework for supporting student well-being in educational facilities. J. Comput. Civ. Eng..

[33] Directive 2002/91/EC of the European parliament and of the council of 16 December 2002 on the energy performance of buildings. *Off J Eur Union* 65e71. 10.1039/ap9842100196 (2002).

[34] Foucquier A, Robert S, Suard F, Stephan L, Jay A (2013). State of the art in building modelling and energy performances prediction: A review. Renew. Sustain. Energy Rev..

[35] Foucquier S, Robert F, Suard L, Stephan A (2013). Jay, State of the art in building modelling and energy performances prediction: A review. Renew. Sustain. Energy Rev..

[36] Runge J, Zmeureanu R (2021). A review of deep learning techniques for forecasting energy use in buildings. Energies.

[37] Fathi S, Srinivasan R, Fenner A, Fathi S (2020). Machine learning applications in urban building energy performance forecasting: A systematic review. Renew. Sustain. Energy Rev..

[38] Chae YT, Horesh R, Hwang Y (2016). An artificial neural network model for forecasting sub-hourly electricity usage in commercial buildings[J]. Energy Build.

[39] Biswas M, Robinson MD, Fumo N (2016). Prediction of residential building energy consumption: A neural network approach [J]. Energy.

[40] Deb C, Eang LS, Yang J (2016). Forecasting diurnal cooling energy load for institutional buildings using artificial neural networks [J]. Energy Build.

[41] Yan K, Li W, Ji Z (2019). A hybrid LSTM neural network for energy consumption forecasting of individual households[J]. IEEE Access.

[42] Zhong H, Wang J, Jia H (2019). Vector field-based support vector regression for building energy consumption prediction[J]. Appl. Energy.

[43] Wang X, Luo D, Zhao X (2018). Estimates of energy consumption in China using a self-adaptive multi-verse optimizer-based support vector machine with rolling cross-validation[J]. Energy.

[44] Tabrizchi H, Javidi MM, Amirzadeh V (2019). Estimates of residential building energy consumption using a multi-verse optimizer-based support vector machine with k-fold cross-validation[J]. Evol. Syst..

[45] Iwafune, Y., Yagita, Y., & Ikegami, T., *et al.* Short-term forecasting of residential building load for distributed energy management[C]. In *2014 IEEE international energy conference (ENERGYCON). IEEE*; pp. 1197–204 (2014).

[46] Albuquerque PC, Cajueiro DO, Rossi MDC (2022). Machine learning models for forecasting power electricity consumption using a high dimensional dataset[J]. Expert. Syst. Appl..

[47] Dong Z, Liu J, Liu B (2021). Hourly energy consumption prediction of an office building based on ensemble learning and energy consumption pattern classification [J]. Energy Build.

[48] Cao W, Yu J, Chao M, Wang J, Yang S, Zhou M, Wang M (2023). Short-term energy consumption prediction method for educational buildings based on model integration. Energy.

[49] Faiq M, Tan KG, Liew CP, Hossain F, Tso CP, Lim LL, Shah ZM (2023). Prediction of energy consumption in campus buildings using long short-term memory. Alex. Eng. J..

[50] Álvarez, J.A., *et al.* Modeling of energy efficiency for residential buildings using artificial neuronal networks. *Adv. Civ. Eng.* (2018).

[51] Beccali M (2017). Artificial neural network decision support tool for assessment of the energy performance and the refurbishment actions for the non-residential building stock in southern Italy. Energy..

[52] Ahmad MW, Mourshed M, Rezgui Y (2017). Trees vs neurons: Comparison between random forest and ANN forhigh-resolution prediction of building energy consumption. Energy Build..

[53] Martellotta F (2017). On the use of artificial neural networks to model household energy consumptions. Energy Proc..

[54] Williams KT, Gomez JD (2016). Predicting future monthly residential energy consumption using building characteristics and climate data: A statistical learning approach. Energy Build..

[55] Sun C, Han Y (2013). Constructing heating energy consumption forecast ANN model for office building in severe cold zone. Architectural.

[56] Wong S, Wan KK, Lam TN (2010). Artificial neural networks for energy analysis of office buildings with daylighting. Appl. Energy.

[57] Catalina T, Virgone J, Blanco E (2008). Development and validation of regression models to predict monthly heating demand for residential buildings. Energy Build..

[58] Alrashed F, Asif M (2014). Trends in residential energy consumption in Saudi Arabia with particular reference to the Eastern Province. J. Sustain. Dev. Energy Water Environ. Syst..

[59] Abdel-Aal RE, Al-Garni AZ, Al-Nassar YN (1997). Modelling and forecasting monthly electric energy consumption in eastern Saudi Arabia using abductive networks. Energy.

[60] Nasr GE, Badr EA, Younes MR (2002). Neural networks in forecasting electrical energy consumption: Univariate and multivariate approaches. Int. J. Energy Res..

[61] Meng M, Shang W, Niu D (2014). Monthly electric energy consumption forecasting using multiwindow moving average and hybrid growth models. J. Appl. Math..

[62] Karatasou S, Santamouris M, Geros V (2006). Modeling and predicting building's energy use with artificial neural networks: Methods and results. Energy Build..

[63] Mena-Yedra, R., Rodriguez, F., Castilla, M. M., & Arahal, M. R. A Neural Network Model for Energy Consumption Prediction of CIESOL Bioclimatic Building. In *International Joint Conference SOCO* (2013)‏.

[64] Somu N, Gauthama Raman MR, Ramamritham K (2020). A hybrid model for building energy consumption forecasting using long short term memory networks. Appl. Energy.

[65] Ullah, F., & Min, U. Short-term prediction of residential power energy consumption via CNN and multilayer bi-directional LSTM networks. *IEEE Access* (2019).‏

[66] Liu B, Chuanchuan Fu (2017). Arlene Bielefield and Yan Quan Liu "Forecasting of Chinese primary energy consumption in 2021 with GRU artificial neural network". Energies.

[67] Khalil, A. J., Barhoom, A. M., Abu-Nasser, B. S., Musleh, M. M., & Abu-Naser, S. S. Energy efficiency predicting using artificial neural network. **3**(9), 1–1 (2019).

[68] Rahman A, Srikumar V, Smith AD (2018). Predicting electricity consumption for commercial and residential buildings using deep recurrent neural networks. Appl. Energy.

[69] Tartibu, L. K., & Kabengele, K. T. Forecasting net energy consumption of South Africa using artificial neural network. In: *2018 International Conference on the Industrial and Commercial Use of Energy (ICUE).* IEEE, pp. 1–7 (2018).‏

[70] Fayaz M, Shah H, Aseere AM, Mashwani WK, Shah AS (2019). A framework for prediction of household energy consumption using feed forward back propagation neural network. Technologies.

[71] Mohammed A, Alshibani A, Alshamrani O, Hassanain M (2021). A regression-based model for estimating the energy consumption of school facilities in Saudi Arabia. Energy Build..

[72] Breiman L (2017). Classification and regression trees.

[73] Peterson LE (2009). K-nearest neighbor. Scholarpedia.

[74] Chen, T., & Guestrin, C. Xgboost: A scalable tree boosting system. In Proceedings of the 22nd acm sigkdd international conference on knowledge discovery and data mining (pp. 785–794) (2016).‏

[75] Graves, A., & Graves, A. Long short-term memory. Supervised sequence labelling with recurrent neural networks, 37–45 (2012).

[76] Uzuner, S., & Çekmecelioğlu, D. Comparison of artificial neural networks (ANN) and adaptive neuro-fuzzy inference system (ANFIS) models in simulating polygalacturonase production (2016).

[77] Sada, S. O., & Ikpeseni, S. C. Evaluation of ANN and ANFIS modeling ability in the prediction of AISI 1050 steel machining performance. Heliyon **7**(2) (2021). 10.1016/j.heliyon.2021.e06136 PMC7856477 33553780

[78] Zhang G, Patuwo BE, Hu MY (1998). Forecasting with artificial neural networks: The state of the art. Int. J. Forecast..

[79] Chong DJS, Chan YJ, Arumugasamy SK, Yazdi SK, Lim JW (2023). Optimisation and performance evaluation of response surface methodology (RSM), artificial neural network (ANN) and adaptive neuro-fuzzy inference system (ANFIS) in the prediction of biogas production from palm oil mill effluent (POME). Energy.

